# Implementation of a Talbot-Lau interferometer in a clinical-like c-arm setup: A feasibility study

**DOI:** 10.1038/s41598-018-19482-z

**Published:** 2018-02-02

**Authors:** Florian Horn, Martino Leghissa, Sebastian Kaeppler, Georg Pelzer, Jens Rieger, Maria Seifert, Johannes Wandner, Thomas Weber, Thilo Michel, Christian Riess, Gisela Anton

**Affiliations:** 1 Friedrich-Alexander-University Erlangen-Nürnberg (FAU), Erlangen Centre for Astroparticle Physics, 91058 Erlangen, Germany; 2 Siemens Healthcare GmbH, 91301 Forchheim, Germany; 3 Friedrich-Alexander-University Erlangen-Nürnberg (FAU), Pattern Recognition Lab, 91058 Erlangen, Germany

## Abstract

X-ray grating-based phase-contrast imaging has raised interest regarding a variety of potential clinical applications, whereas the method is feasible using a medical x-ray tube. Yet, the transition towards a clinical setup remains challenging due to the requirement of mechanical robustness of the interferometer and high demands applying to medical equipment in clinical use. We demonstrate the successful implementation of a Talbot-Lau interferometer in an interventional c-arm setup. The consequence of vibrations induced by the rotating anode of the tube is discussed and the prototype is shown to provide a visibility of 21.4% at a tube voltage of 60 kV despite the vibrations. Regarding clinical application, the prototype is mainly set back due to the limited size of the field of view covering an area of 17 mm × 46 mm. A c-arm offers the possibility to change the optical axis according to the requirements of the medical examination. We provide a method to correct for artifacts that result from the angulation of the c-arm. Finally, the images of a series of measurements with the c-arm in different angulated positions are shown. Thereby, it is sufficient to perform a single reference measurement in parking position that is valid for the complete series despite angulation.

## Introduction

In clinical routine, up to now solely the attenuation of x-rays interacting with matter has been employed to form the image. In addition, a variety of promising phase-sensitive methods evolved, which rely on the refraction of x-rays^1,2^. Yielding improved soft tissue contrast^3^, a major shortcoming of conventional attenuation-based imaging, those methods have been extensively investigated with regard to medical applications^4–6^. Among others, x-ray grating-based phase-contrast imaging^7–9^ is a imaging modality that has been expanded to medical tubes of low brilliance^10^, as well. Simultaneously, a conventional attenuation image, a differential phase-contrast image, and a dark-field image is calculated from a reference measurement without object and an object measurement with the object in place. Thereby, the dark-field image provides complementary information about the subpixel-size structures of a granular object^11–15^, whereas information about the orientation of the substructure is included^16–18^.

The method raised interest due to a number of promising medical applications, such as mammography^19–21^, lung imaging^22–24^, and imaging of the joints^25–27^. Scaling the method from laboratory setups to human-size specimen in a preclinical setup, several challenges have to be overcome: the transition towards higher x-ray energies suitable for medical imaging of larger parts of the human body, the increment of the size of possible measurable specimens, and the realization of sufficient image quality at low doses to enable *in vivo* imaging in combination with fast data acquisition.

Regarding medical applications carried out using a medical x-ray tube with large focal spot size and a polychromatic x-ray spectrum at higher acceleration voltages above 50 kV, a first measurement has been reported with a CT measurement of a human cervical spine^28^. Recently, a radiographic measurement of a human knee has been published revealing signatures of chondrocalcinosis of the medial meniscus applying a dose that is comparable to conventional attenuation-based devices^29^. The tube voltage has been 70 kV for both measurements.

While typical sizes of the field of view have been in the range of a few centimeters, recently progress towards larger specimens has been reported by either increasing the grating sizes up to 200 mm × 200 mm by stitching of smaller gratings^30–32^ or the realization of scanning radiographic devices^33–35^, which means that the object is scanned by an interferometer with sufficient size to capture the object at once in one dimension. Additionally, the transition to a scanning setup overcomes the time-consuming phase-stepping procedure^9^, as the phase-stepping curve can then be measured by continuously moving the object through the scanner, if the phase image of the reference measurement comprises at least a complete fringe of the Moiré pattern in scanning direction^36^. A scanning interferometer has also been reported for an edge-illumination setup, recently^37,38^.

*In vivo* measurement of small animals has been demonstrated for a radiographic measurement by Bech *et al*.^39^ using a multiple image small-animal scanner operated at 31 kV tube voltage, which proved that the retrieval of the differential phase-contrast and dark-field image is still possible despite the object motion. The realization of a gantry-type small-animal CT-setup and its challenges towards Talbot-Lau imaging have been described by Tapfer *et al*.^40^. As an alternative to conventional iodine-based angiography, the application of microbubbles commonly used in ultrasound imaging has been reported as a scattering contrast agent in the dark-field image for Talbot-Lau imaging^41^ and diffraction enhanced imaging^42,43^. Regarding Talbot-Lau imaging, the *ex vivo* CT-measurement aiming at the depiction of the microbubble contrast agent has been performed using the same small-animal CT-setup as an additionally reported *in vivo* CT-measurement of mice by Velroyen *et al*.^44^. Further small-animal studies comprise imaging of emphysema^45^, pneumothoraces^46^, and lung cancer^47^ of murine lungs.

With the report of an *in vivo* dark-field chest radiography of a pig’s lung carried out at 70 kV within 40 s by Gromann *et al*.^34^ a major milestone, covering all of the aforementioned demands, has been achieved. Thereby, the applied dose of 80 μSv for a human-sized specimen is clinically acceptable indicating the possible transition from “bench to bedside”.

However, signal extraction remains challenging taking in mind the small grating periods, typically in the range of a few microns. Hence, the mechanical robustness of the interferometer is key together with sophisticated image retrieval algorithms that correct for artifacts caused by mismatches in grating alignment^40^ and phase-step position^48–51^. An alternative to phase-stepping is especially important for CT-measurements, as a continuously rotating gantry is a requirement for clinical applications. Recently, a robust data acquisition protocol that reduces the necessary number of measurements to a single image for each projection has been published^52,53^, which represents important progress.

Regarding the realization of setups that aim at clinical imaging, a prototype dedicated to the diagnosis of rheumatoid arthritis in human finger joints has been reported by Tanaka *et al*.^54^ and Momose *et al*.^55^ which is operated *in vivo* in a clinical environment. Thereby, the field of view is restricted to 49 mm × 49 mm, though, and the dose is quite high with about 5 mGy at a limited tube voltage of 40 kV. Nevertheless, the depiction of cartilage confirms the clinical potential. Additionally, considerations with regard to the development of a device aimed at mammography have been summarized by Roessl *et al*.^56^ which led to the development of a slit-scanning mammography device^33^. Further investigations for the optimization of interferometer specifications aiming at a clinical mammography setup have been reported^57^. Experimental studies have been carried out by Li *et al*.^58^ concerning a tomosynthesis setup.

In general, additional restrictions apply to a clinical Talbot-Lau imaging device in comparison to a laboratory setup. The interferometer’s length is restricted for practical reasons in a hospital and because of the quadratic decline of the flux with increasing distance from the source, which leads to an intolerable boost of exposure time. A vertical oriented optical axis is favorable regarding patient positioning and the interferometer can’t be operated on an optical table, which yields a disturbance of the measurement by mechanical vibrations and may cause the appearance of artifacts due to unstable Moiré fringes. Of course, the entailing costs of a transition towards the clinics have to be held at a bearable amount, as well.

Altogether, these reasons favor the implementation of a Talbot-Lau interferometer in a yet existing clinical device. An interferometer designed to operate at high tube voltages between 50 kV and 125 kV has been mounted on a c-arm identical to a clinical system and tested for feasibility regarding Talbot-Lau imaging.

With the possibility to angulate the c-arm arm and thus change the optical axis around a patient lying on the table, a c-arm offers high flexibility in interventional medical imaging. Thereby, the inherent restrictions with regard to mechanical stability of a c-arm, the medical tube with a rotating anode, which induces vibrations to the c-arm and gratings, and mechanical deformation of the interferometer provide a challenging scenario for interferometric imaging. Experimental studies have been performed to assess the feasibility of the prototype system.

## Results

### Demonstration of feasibility

**Figure 1:**
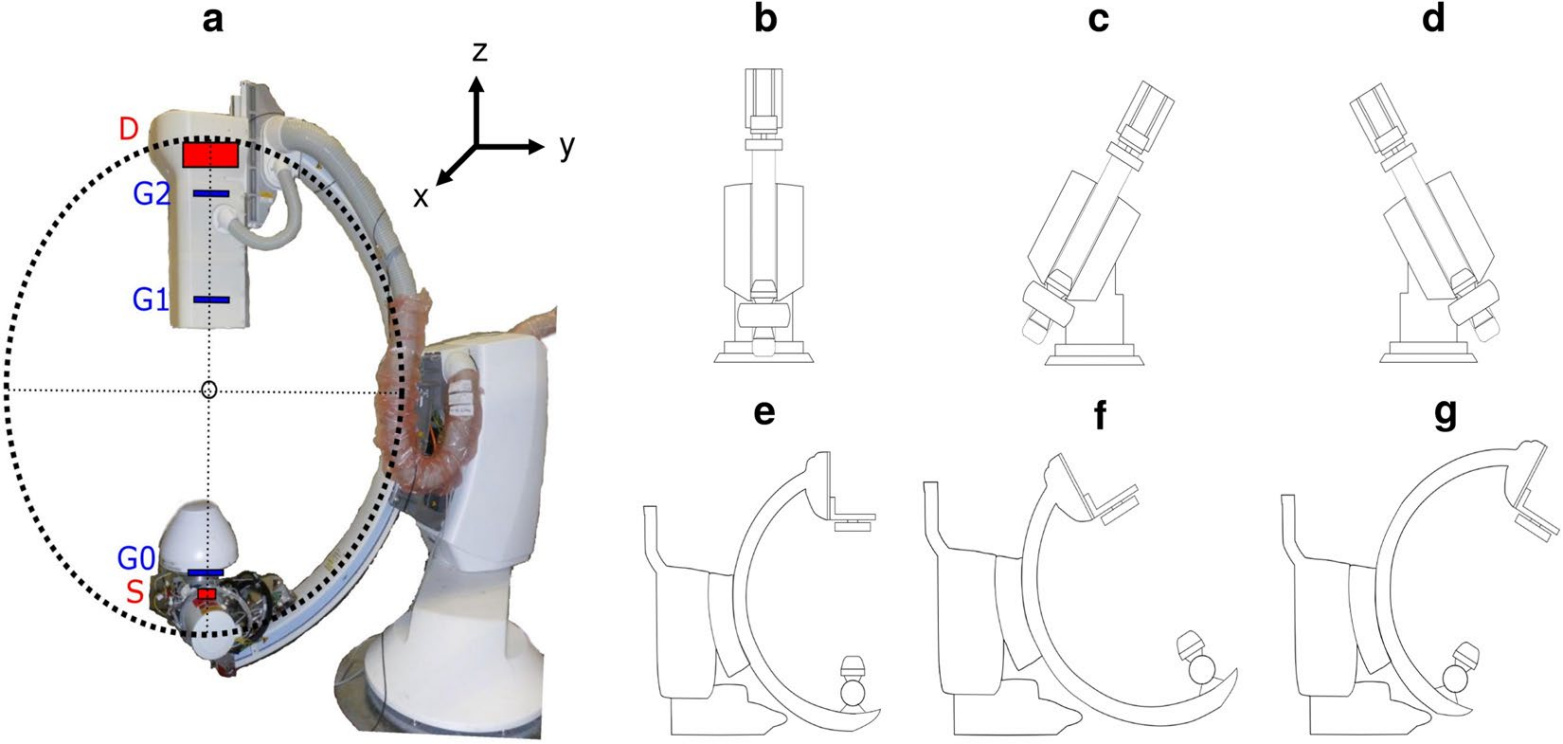
**(a)** C-arm Talbot-Lau prototype based on Siemens Healthineers Artis one floor-mounted system. **(b)** Sketch of frontal view of a c-arm in parking position. **(c)** Sketch of frontal view of a c-arm in 30° LAO-angulation. **(d)** Sketch of frontal view of a c-arm in 30° RAO-angulation. **(e)** Sketch of frontal view of a c-arm in parking position. **(f)** Sketch of side view of a c-arm in 30° CRAN-angulation. **(g)** Sketch of side view of a c-arm in 30° CAUD-angulation.

A sketch of the used c-arm prototype is shown in Fig. 1(a). A regular Talbot-Lau interferometer has been used consisting of a polychromatic medical x-ray tube with an extended focal spot *S*^10^, a flat-panel scintillating x-ray detector *D*, and the three gratings *G*0, *G*1, and *G*2. The Talbot-Lau interferometer has been mounted to a conventional clinically used c-arm floor-mounted angiography setup. A c-arm platform can be used flexibly in a variety of medical imaging and surgical procedures, as it features the possibility to take images from a broad range of angles^59^. The used c-arm offers two independent modes of angulation, which differ in the direction of motion of the detector from the perspective of the patient. The detector can be moved in cranial/caudal (CRAN/CAUD) direction of rotation, as well as in left/right anterior oblique (LAO/RAO) direction. Thereby, a cranial/caudal angulation means that the c-arm is angulated in such a way that the direction of propagation is changed head-wards/foot-wards. On the contrary a left/right anterior oblique rotation means that the x-rays are emitted from the right/left hand side towards the left/right hand side of the patient lying on the table. Figure 1(b–g) illustrates the different modes of angulation.

The c-arm device works in conjunction with the patient table which can be moved to a suitable height and position, therefore increasing the flexibility of the device. In the case of Talbot-Lau imaging, the patient should be moved as close as possible to *G*1 to maximize sensitivity^60,61^.

**Figure 2:**
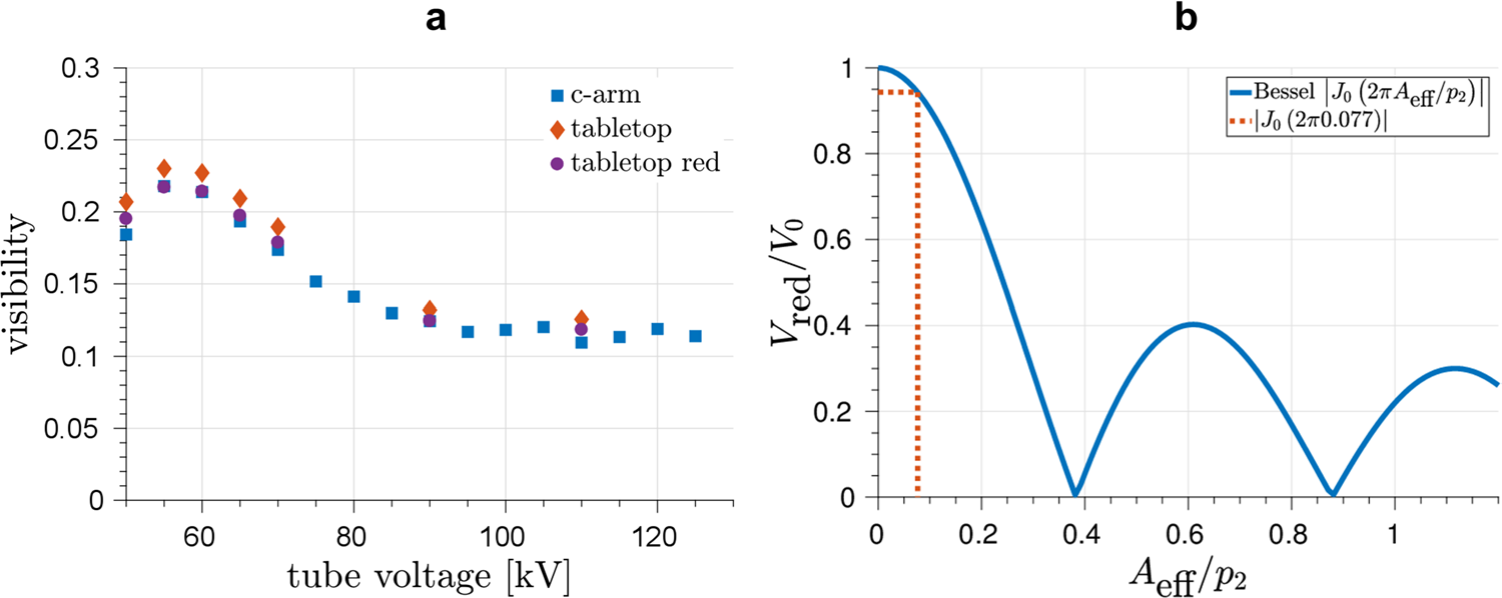
**(a)** Measured mean visibilities over the field of view for various acceleration voltages for both the interferometer mounted to the c-arm and placed in a laboratory tabletop setup. Additionally, calculated visibilities applying the visibility reduction model to the measured visibilities of the tabletop setup are shown. **(b)** Relative reduction of the visibility due to high-frequency vibrations of the gratings as a function of the oscillation amplitude relative to *p*_2_. The red dashed line marks the worst case scenario of the introduced model assuming *A*_eff_/*p*_2_ = 0.077, as described in section methods.

For reasons of comparison, the mean visibilities achieved by the Talbot-Lau interferometer used have been determined for various tube voltages with the interferometer consisting of the same gratings either mounted to the c-arm or placed on an optical table. The result is given in Fig. 2(a) for tube voltages between 50 kV and 125 kV. At 60 kV, where the measurements have been carried out, the mean visibility met 21.4%. Both setups show a similar behavior, whereas the visibilities for the tabletop setup slightly surpasses the c-arm. A reduction of the visibility for the c-arm setup is expected due to high-frequency vibrations of the gratings. This effect can be quantified by an analytical model resulting in Eq. 5, which is described in the methods section in more detail. The differences between the measured mean visibilities of both setups fall within 2% in most cases, which is expected according to the model. Additionally, the calculated reduced visibilities applying the model to the measured visibilities of the tabletop setup are drawn for comparison.

Figure 2(b) shows the calulated relative reduction of the visibility due to high-frequency vibrations depending on the effective oscillation amplitude *A*_eff_ in relation to the analyzer grating period *p*_2_. Thereby, *A*_eff_ is determined by the oscillation amplitudes and the phase between the oscillations of the source grating and the phase grating. The oscillation amplitudes of both gratings have been measured to be below 0.2 μm in phase-stepping direction. A worst case scenario with the two vibrations being opposite in phase is marked by the red dashed line for *A*_eff_/*p*_2_ = 0.077 and reveals a relative reduction of 5.8%, which yields 1.3% as an absolute reduction for a visibility of 22.7% of the tabletop setup at 60 kV. Thereby, the calculated reduction matches the gap between both measured mean visibilities. A realignment of the interferometer and the variance of measured visibilities may yield an additional offset of the measured visibilities, which is of a similar scale, though.

**Figure 3:**
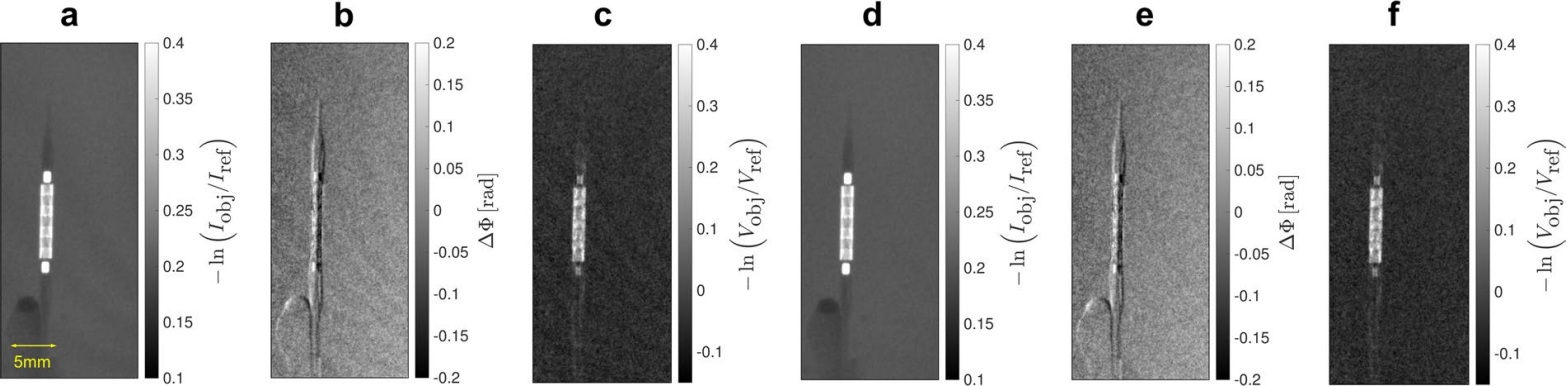
Non-inflated stent with a dilatation catheter placed in a water bath with a height of 13 mm. The dose has been 0.54 mGy measured as air kerma. **(a**–**c)** From left to right: attenuation, differential phase-contrast, and dark-field image using a standard reconstruction algorithm applying a Fourier transform with respect to the phase-steps. Due to phase drift effects Moiré fringe artifacts are visible in the images. **(d–f)** From left to right: Attenuation, differential phase-contrast, and dark-field image of the stent using an advanced reconstruction algorithm adjusting the phase-step positions. The algorithm is able to remove the fringe artifacts.

As a demonstration of feasibility, a non-inflated stent with a dilatation catheter has been placed in a water bath with a height of 13 mm. The stent of type Cordis Presillion is made of cobalt-chromium (CoCr) and features both cells with wide (60–70 μm) and narrow (30–35 μm) strut thicknesses^62^. It has an non-inflated length of 9 mm, while the diameter measures 1.78 mm. Due to inflation it expands to a diameter of 4 mm. The images are included in Fig. 3(a–f) applying two different reconstruction algorithms.

Displaced gratings cause a shift of the Moiré pattern yielding a deviation from the intended position while measuring a phase-step and lead to the formation of fringe artifacts in the reconstructed images. Thereby, the phase-drift effects are caused by thermal influences on the interferometer and mechanical instabilities of the c-arm, which change the imprinted Moiré pattern on a time scale that is longer than the time of measurement of a single phase-step. With the source grating *G*0 being integrated in the collimator next to the tube source the interferometer is subject to heating of the tube. Figure 3(a–c) shows the resulting images using a standard reconstruction method applying a Fourier transform with regard to the phase-step positions giving rise to the formation of Moiré artifacts. On the contrary, Fig. 3(d–f) proves the removal of the artifacts using an improved reconstruction algorithm, which adjusts the phase-step positions^51^.

The images reveal that the non-inflated stent is clearly visible in the dark-field image due to the sharp edges of the mesh. This result can be explained by the additionally generated visibility contrast for objects with unresolvable edges in the differential phase-contrast image, as shown by Yashiro and Momose^63^. Concerning the attenuation image, the highest signal is measured for the radiopaque markers made of platinum iridium, whereas in the dark-field image the stent struts themselves cause the highest signal. The dose of the measurement has been 0.54 mGy measured as air kerma.

### Angulation

The core feature of a c-arm device is the possibility to angulate the c-arm meaning a change of the optical axis from the x-ray source to the detector. The normal position of a c-arm is with a vertically oriented optical axis, labeled as parking position. By angulation, a mechanical shake is caused to the c-arm and the c-arm is deformed due to a change in the impact of gravity. The deformation provokes a misalignment of the interferometer. Hence, the shape of the Moiré pattern is changed and the visibility is reduced.

**Figure 4:**
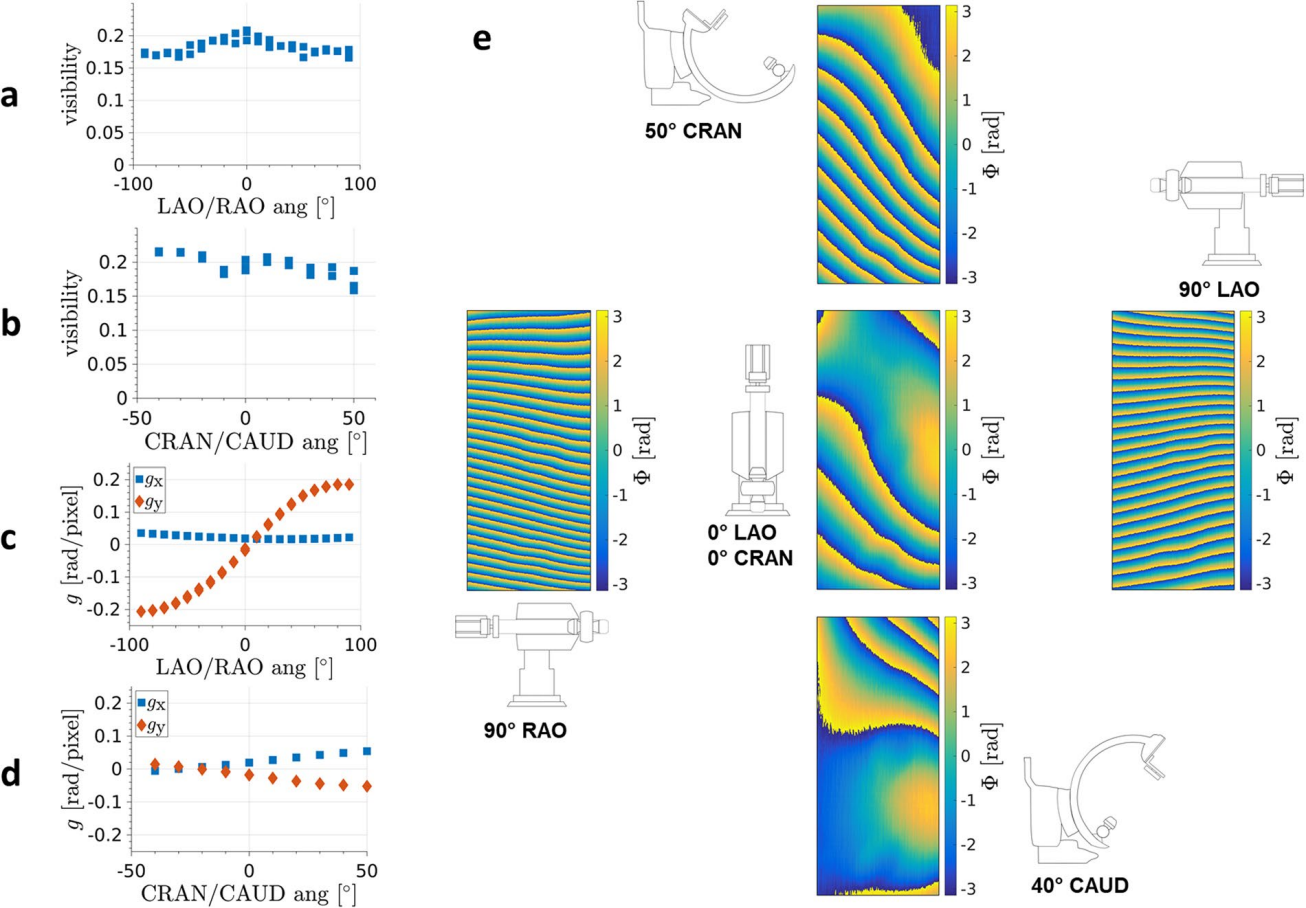
**(a)** Measured mean visibilities for a LAO-/RAO-angulated c-arm depending on angulation angle. Positive angles correspond to LAO-angulation. **(b)** Measured mean visibilities for a CRAN-/CAUD-angulated c-arm depending on angulation angle. Positive angles correspond to CRAN-angulation. **(c)** Gradients of the fitted polynomial of first degree to the phase ramps created by LAO-/RAO-angulation. Positive angles correspond to LAO-angulation. **(d)** Gradients of the fitted polynomial of first degree to the phase ramps created by CRAN-/CAUD-angulation. Positive angles correspond to CRAN-angulation. **(e)** Visualization of phase distributions of reference measurements for various angulated positions of the c-arm.

In order to assess the feasibility of measurements with the c-arm in an angulated position, a series of reference measurements has been carried out with the c-arm driven to various positions. Thereby, the interferometer has not been realigned after angulation and the phase-stepping curves have been measured after a pause of 1–3 s to allow for a relaxation of vibrations caused by angulation. Figure 4(a) shows the measured mean visibilities over the field of view for a LAO-/RAO-angulated c-arm. A decrease is visible towards higher angulation angles. It is evident that the decrease slows down for angles higher than 50° for both LAO- and RAO-angulation. Thereby, the maximum reduction is caused from 21% to 17% for 90° in both directions. The mean visibilities of CRAN-/CAUD-angulation are displayed in Fig. 4(b). Towards higher angles in caudal direction even a slight increase of mean visibility is noticeable, which means that the interferometer has not been adjusted to an optimal state in the parking position. With a difference of 1% in visibility compared to the parking position this offset is minor, though. Altogether, the mean visibilities comprise values from 16% to 22% at 60 kV tube voltage.

In addition to the influence on visibility, angulation changes the phase distribution, whereas a misalignment of the interferometer causes an increase of the frequency of the Moiré pattern. Figure 4(e) visualizes the phase distributions of reference measurements for various angulated positions of the c-arm comprising a value range from −*π* to *π*. If the position of the c-arm differs in between a reference and an object measurement, the mismatch in the reference phase distribution overlays the actual differential phase-contrast image formed by the specimen. This artifact can be corrected by an algorithm based on fitting a polynomial of first degree to the unwrapped phase images. The method has been described by Seifert *et al*.^49^ in more detail applying a fit of polynomials of higher degree to correct for instable reference phase distributions over time. Figure 4(c) shows the gradients of the polynomial for LAO-/RAO-angulation and (d) for CRAN-/CAUD angulation. The highest gradients have been determined for a c-arm angulated by 90° in LAO- or RAO-direction with a gradient exceeding 0.2 rad/pixel in y-direction, which means in the direction perpendicular to the phase-stepping. As the Moiré fringe pattern is created by line gratings along the y-axis and the fringe pattern arises in a perpendicular direction, the theory of Moiré effect indicates a rotation of the gratings in relation to each other around the optical axis^64^. The gradients in x-direction are less pronounced. A corrected differential phase-contrast image is then given by the difference of the residuals of the object and reference measurement eliminating the phase ramps.

### Correction of artifacts resulting from angulation

**Figure 5:**
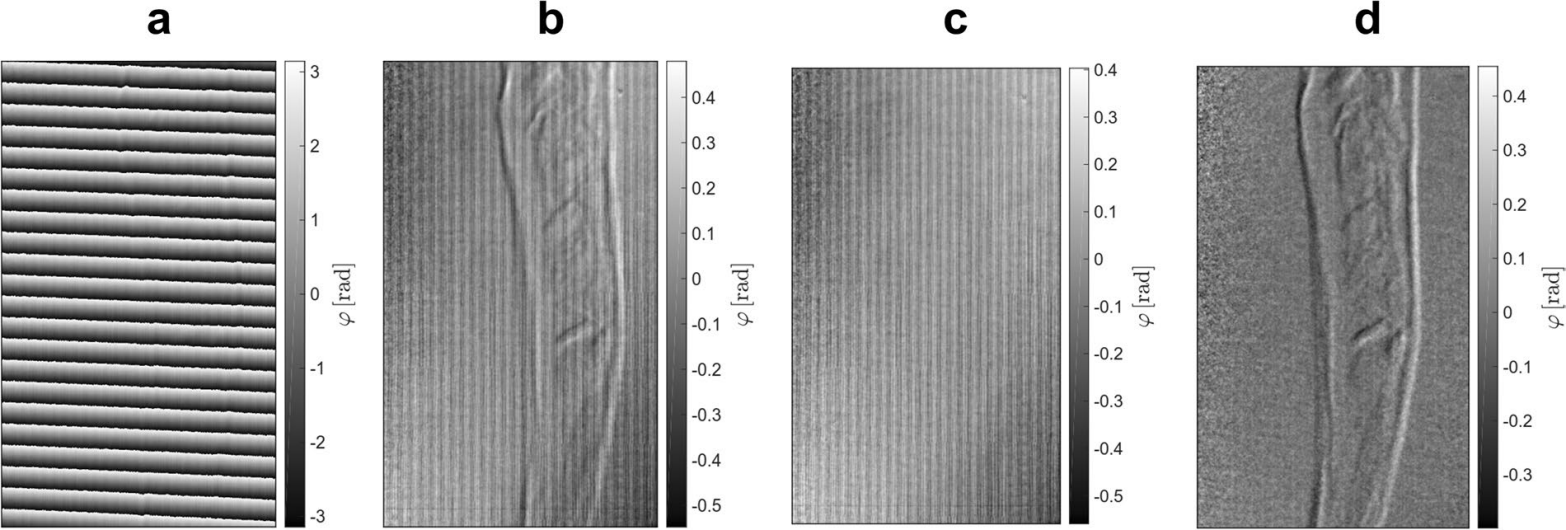
**(a)** Differential phase-contrast image calculated with the c-arm in the parking position for the reference measurement and in 50° RAO-angulated position for the object measurement. **(b)** Compared to (a) the ramp artifacts have been removed by subtraction of the fitted polynomials to the reference and object measurement. The image is calculated by the difference of the residuals. Artifacts due to displaced grating structures are still visible. **(c)** Calibration image calculated by the difference of the residuals of two reference measurements carried out in the parking position and in 50° RAO-angulated position. **(d)** Final corrected image.

Angulation leads to a misalignment of the gratings, which gives rise to a reduction of visibility and causes an increase in the frequency of the Moiré pattern. If a reference measurement and a measurement with the object in place are carried out at different angulated positions of the c-arm, the phase shift remains visible in the differential phase-contrast image deteriorating image quality, which is shown in Fig. 5(a). Therefore, without any further post-processing a new reference measurement would be an obligation after each movement of the c-arm. In clinical routine, this requirement would reduce the possible throughput and potentially prevent the use of a Talbot-Lau c-arm in a medical procedure.

Hence, we propose additional post-processing steps to maintain image quality in the differential phase-contrast image. Firstly, a 2-D Constantini-unwrapping^65^ is performed to the phase image of both the reference and object measurement. Afterwards, a polynomial of first degree according to Φ(*x*_pixel_,*y*_pixel_) = Φ_0_ + *g*_x_ ⋅ *x*_pixel_ + *g*_y_ ⋅ *y*_pixel_ is fitted to the unwrapped data. A preliminary differential phase-contrast image can then be determined by calculating the difference between the residuals of both fits. In doing so, Fig. 5(b) proves the removal of the phase ramps due to angulation. This approach is similar to the adaptive differential phase recovery method described by Tapfer *et al*.^40^ and identical to the image reconstruction routine described by Seifert *et al*.^49^. However, after subtraction of the fitted polynomial from a phase image the residuals still contain information about grating structures and their position relative to the detector pixels. As the residuals of reference measurements carried out at different angulations were found to be different, a calibration measurement consisting of reference measurements for every possible angulation angle is performed prior to the actual imaging procedure. Afterwards, the difference of the residuals is calculated for every possible combination and saved for post-processing. Exemplary, the calibration image consisting of the difference of two residuals of reference measurements with the c-arm in the parking position and in 50° RAO-angulated position is shown in Fig. 5(c). Since residuals for reference measurements at the same angulated position of the c-arm were found to be stable despite an intermediate movement of the c-arm, the difference of the calibration measurement can be subtracted from the difference of the actual object and reference measurement yielding the final corrected differential phase-contrast image depicted in Fig. 5(d).

As the mean visibility, the distribution of visibilities and the contribution of the specimen holder to measured attenuation changes with angulation, a similar prior calibration measurement has been performed for the attenuation and dark-field image, as well. Thereby, the attenuation and dark-field images have been calculated between reference measurements with the c-arm in parking position and in different angulated positions. Later-on the calibration images can be subtracted from the uncalibrated images without the necessity of subtracting fitted polynomials in a previous step.

### Demonstration of a specimen measurement for different angulated c-arm states

A proof-of-concept measurement has been executed measuring a chili from different angulation angles and applying the proposed image correction algorithm. Even though not representing a clinical application, the sample is sufficient to examine the robustness of the setup and the proposed algorithm. As the residuals of two reference measurements from different angulations disagree, indicating a shift of grating structures in relation to the pixel matrix, the proposed calibration measurement has been carried out consisting of a series of reference measurements with the c-arm driven to every position. Afterwards, the c-arm has been re-positioned in the parking position and the actual reference measurement has been performed, which is used to calculate the three images of Talbot-Lau imaging. Finally, the object measurements have been carried out at the different angulated positions of the c-arm without measuring further reference measurements. Hence, the c-arm has been angulated in between every object measurement and the used reference measurement.

**Figure 6:**
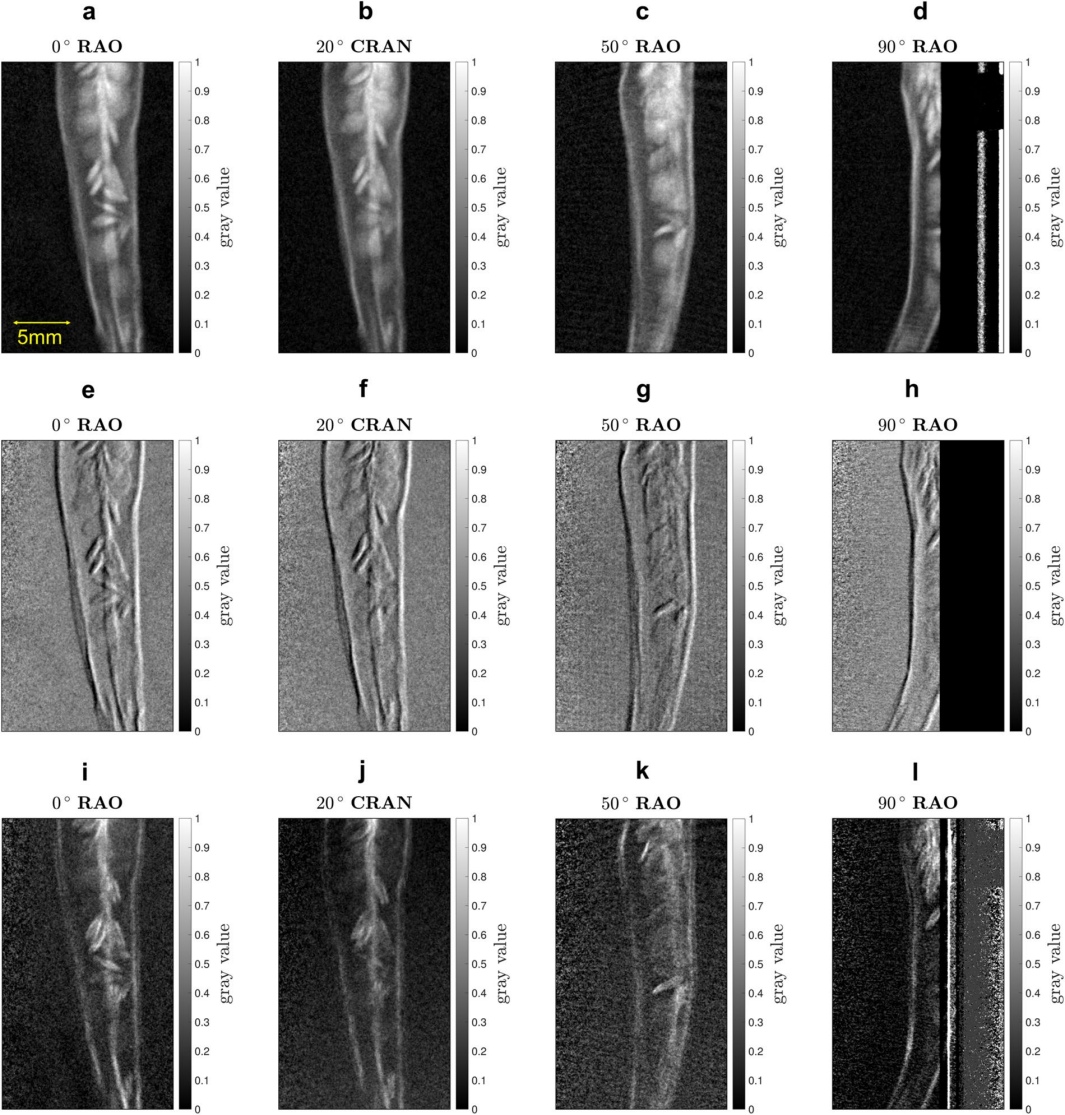
Measurement of a chili for different projections angulating the c-arm. Each measurement has been carried out at a dose level of 0.36 mGy measured as air kerma. For all images the described correction algorithm has been used. A single reference measurement was carried out with the c-arm in the parking position (0° RAO). The position of the c-arm is given by the title. **(a–d)** Attenuation image. **(e–h)** Differential phase-contrast image. **(i–l)** Dark-field image.

As an example, Fig. 6(a–d) show the resulting attenuation, (e)–(h) the differential phase-contrast, and (i)–(l) the dark-field images. For the measurement at 90° RAO-angulation, the specimen holder made of metal came in the field of view, which is visible in the right part of the images. The images display only a minor amount of artifacts proving the successful application of the correction algorithm and the feasibility of Talbot-Lau interferometry using a clinical c-arm setup, which is angulated in between the reference and object measurement. Thereby, the dose has been 0.36 mGy for each projection, measured as air kerma.

## Discussion

The measurements prove the successful implementation of a Talbot-Lau interferometer in a clinically used c-arm angiography setup. The prototype reached a mean visibility of 21.4% at a tube voltage of 60 kV and sufficient visibility up to 125 kVp to perform Talbot-Lau imaging. A method to estimate the reduction of visibility due to high-frequency grating vibrations has been introduced and experimentally validated. An improved reconstruction algorithm adjusting phase-step positions has been tested for a setup being subject to phase-drift effects to a greater extent in comparison to a laboratory setup. The algorithm was shown to be able to remove visible Moiré artifacts.

The setup was found to be mechanically robust against deformation caused by angulation due to only a minor reduction of the visibility. Hence, a measurement without a realignment of the interferometer after angulation is feasible. Artifacts formed by a misalignment of the gratings could be successfully corrected by the applied algorithm, allowing for repeated object measurements at different c-arm positions without the need for a new reference measurement after every angulation. This is a requirement for a potential clinical application of a Talbot-Lau interferometer mounted to a c-arm.

We experienced a shift of grating structures in relation to the pixel matrix caused by a movement of the gratings relative to the detector. In the constructive design of future prototypes a more proper fixation of the gratings is necessary, as even slight deviations are visible when comparing measurements with an intermediate movement of the setup.

The usability of the prototype is hold back by its grating sizes. In fact, the additional restriction due to shadowing further limits the size of the field of view to 17 mm × 46 mm, which is too small for most clinical applications. However, shadowing can be corrected by bending of the gratings^66,67^ and recently the advance towards larger grating sizes has been reported by stitching of smaller gratings^31,32^. Both, bent gratings and the usage of larger gratings, can be transferred from a laboratory setup to a c-arm platform. Therefore, in a next step towards the clinics, recent progress with regard to the ability of measuring larger specimens due to larger gratings or a scanning approach^34,35,38^ at a time span that allows for *in vivo* measurement has to be combined with medical equipment, e.g. a gantry, a c-arm or a conventional radiography system. In addition, for an angiography system methods for real-time fluoroscopic phase-contrast imaging need to be developed.

The doses applied have been 0.54 mGy for the measurement of the stent and 0.36 mGy for the measurement of the chili. Considering the size of the samples, the doses are higher compared to clinical radiographs. However, the parameters have been chosen to examine the mechanical robustness of the setup without further optimization regarding dose. In general, compared to a laboratory setup the same considerations with regard to dose apply for the prototype with except of the small reduction of visibility due to high-frequency vibrations, which results in increased noise^68,69^. Recently, two publications reported measurements carried out at a dose level that is comparable to the clinics^29,34^. Using the same interferometer specifications, the findings could be transferred to a c-arm platform.

The successful implementation of a grating-based phase-contrast imaging setup in a clinical device proving sufficient mechanical robustness for interferometric imaging represents progress in the research aiming at the translation towards clinical practice.

## Methods

### Talbot-Lau interferometry

A regular Talbot-Lau interferometer has been used consisting of a polychromatic medical x-ray tube with an extended focal spot *S*^10^, a flat-panel scintillating x-ray detector *D*, and three gratings. The phase grating *G*1 imprints a periodical phase-shift to the wave-front produced by the x-ray tube, which leads to a downstream intensity fringe pattern matching the shape of *G*1 at certain distances due to the Talbot effect. In addition, the source grating *G*0 is needed to satisfy the requirements regarding spatial coherence by a partition of the focal spot in several slit sources each fulfilling the coherence requirement and producing its own intensity pattern. As the slit sources are mutually incoherent, the parameters of *G*0 have to be chosen leading to matching positions of each intensity pattern. An object in the beam path modifies the intensity pattern and may lead to a reduction of the mean intensity, a local change of the position of the fringes caused by deflection and an alternation of the amplitude of the fringes. Accordingly, the attenuation image, differential phase-contrast image, and the dark-field image can be calculated by a comparison of a reference measurement without object and an object measurement with the object in place. As the period of the intensity pattern typically is below the pixel pitch of the detector, an additional analyzer grating *G*2 is used to sample the intensity pattern by a phase-stepping technique^9^. The resulting phase-stepping curve in dependence of the phase-step position *x* measured for each pixel is described by

1 $$I(x)={I}_{0}[1+{V}_{0}\,\sin (\frac{2\pi }{{p}_{{\rm{G}}2}}x+\varphi )],$$

where *I*_0_ is the mean intensity, *V*_0_ the visibility defined by *V*_0_ = (*I*_max_ − *I*_min_)/(*I*_max_ + *I*_min_), and Φ the local offset of the position. The attenuation image Γ, the differential phase-contrast image ΔΦ, and the dark-field image Σ can then be calculated according to

2 $$\begin{array}{ccc}{\rm{\Gamma }}=-\mathrm{ln}(\frac{{I}_{0}^{{\rm{obj}}}}{{I}_{0}^{{\rm{ref}}}}), & {\rm{\Delta }}{\rm{\Phi }}={{\rm{\Phi }}}_{{\rm{obj}}}-{{\rm{\Phi }}}_{{\rm{ref}}}, & {\rm{and}}\,\,{\rm{\Sigma }}=-\mathrm{ln}(\frac{{V}_{0}^{{\rm{obj}}}}{{V}_{0}^{{\rm{ref}}}}).\end{array}$$

### Description of the setup

The Talbot-Lau interferometer has been mounted to a Siemens Artis one, which is a conventional clinically used floor-mounted c-arm angiography setup. The *G*0 grating has been integrated between X-ray source and collimator. A unit consisting of the *G*1, *G*2 grating and detector is replacing the conventional detector. The interferometer consisted of three gratings manufactured by Microworks: the source grating *G*0 with the period *p*_0_ = 11.54 μm and the height *h*_0_ = 270 μm, the phase grating *G*1 with the period *p*_1_ = 3.39 μm and the height *h*_1_ = 6.37 μm imprinting a phase shift of *π*/2 at the grating bars at the design energy of 62.5 keV, and the analyzer grating *G*2 with the period *p*_2_ = 4.8 μm and the height *h*_2_ = 160 μm. All grating have been made of gold with a duty cycle of 0.5. *G*1 has been placed *l* = 986 mm away from *G*0. The distance between *G*1 and *G*2 amounted to *d*_T_ = 410 mm.

As x-ray source a medical x-ray tube of type Siemens Megalix CAT Plus 125/40/90 with a rotating anode made of tungsten has been used, which can be driven at acceleration voltages between 40 kV and 125 kV. The focal spot size has been 0.4 (IEC 60336), respectively 0.6 mm × 0.85 mm (H×V) at 15% of the maximum value. The x-rays have been detected using an integrating flat panel detector PerkinElmer Dexela 1512 with 74.8 μm pixel size and a 600 μm thick CsI scintillation layer. The x-ray spectrum has been filtered with a 0.3 mm thick copper plate to suppress x-ray photon energies below 30 keV.

The images have been acquired at 60 kV tube voltage reaching a visibility of 21.4±1.8% over the field of view. The size of the field of view at the position of *G*1 has been restricted to 17 mm × 46 mm due to shadowing in phase-stepping direction. Every phase-stepping curve has been measured with a tube current of 50 mA. The number of phase-steps and the acquisition time of each phase-step varied between the measurements. The chili has been measured at 17 phase-step positions with an acquisition time of 250 ms per phase-step. The measurement of the stent has been performed at 32 positions with an acquisition time of 200 ms per phase-step.

### Reduction of visibility due to high-frequency grating vibrations

The x-ray tube with a rotating anode is mounted to the c-arm. In consequence, vibrations are transferred to the c-arm and the fixed interferometer. Measurements regarding the amplitude and frequency of occurring vibrations with an operating tube have been carried out using triaxial piezoelectric accelerometers, which revealed that vibrations are mainly originating from the anode rotating with a frequency of 156 Hz. The acceleration amplitudes at gratings are typically ≤200 mm/s^2^ corresponding to displacement amplitudes of about 0.2 μm in phase-stepping direction for a frequency of 156 Hz. Consequently, as the vibration period is much shorter than the acquisition time for one phase step the vibrations lead to a reduction of the visibility due to smearing of the phase-stepping curves. The reduction of the visibility can be quantified according to the following considerations.

The visibility *V*_0_ is usually given by *V* = (*I*_max_ − *I*_min_)/(*I*_max_ + *I*_min_) with *I*_max_ and *I*_min_ being the intensity at the maximum, respectively the minimum of the phase-stepping curve. The phase-stepping curve itself is described by Eq. 1. Due to the grating vibrations *x* = *x*(*t*) is depending on the time *t*. It is assumed that the analyzer grating’s position, which samples the phase-stepping curve, is fixed and the intensity pattern created by the gratings *G*1 and *G*2 performs a sinusoidal oscillation caused by the vibrations of both gratings. The position of the intensity pattern then changes over time according to

3 $$x(t)={x}_{0}+{A}_{{\rm{eff}}}\,\sin (\frac{2\pi }{T}t+{\varphi }_{{\rm{t}}})\mathrm{.}$$

As the initial position of the phase-stepping curve is arbitrary regarding visibility, the phase *ϕ* = 0 may be set to zero without a loss in generality. With *t*_acq_ being the acquisition time of each phase-step it is assumed $T\ll {t}_{\mathrm{acq}}$. Hence, the phase *ϕ*_t_ = 0 is neglected and the intensity pattern is expected to perform an integer amount of repetitions of the oscillation. Consequently, the average intensity of the phase-step ${\hat{I}}_{\max }$ at the maximum position *x*_max_ = *g*_2_/4 is given by

4 $$\begin{array}{rcl}{\hat{I}}_{{\rm{\max }}} & = & \frac{1}{T}{\int }_{0}^{T}{I}_{0}[1+{V}_{0}\,\sin (\frac{2\pi }{{p}_{G2}}({x}_{{\rm{\max }}}+{A}_{\mathrm{eff}}\,\sin (\frac{2\pi }{T}t)))]{\rm{d}}t\\  & = & {I}_{0}+{I}_{0}{V}_{0}\,{J}_{0}(2\pi \frac{{A}_{{\rm{eff}}}}{{p}_{{\rm{G}}2}}),\end{array}$$

where *J*_0_ is the Bessel function of the first kind. Similar calculations result in ${\hat{I}}_{{\rm{\min }}}={I}_{0}-{I}_{0}{V}_{0}\,{J}_{0}[(2\pi {A}_{{\rm{eff}}})/{p}_{{\rm{G}}2}]$. In summary, the relative reduction of the visibility by the oscillations using ${V}_{{\rm{red}}}=({\hat{I}}_{{\rm{\max }}}-{\hat{I}}_{{\rm{\min }}})/({\hat{I}}_{{\rm{\max }}}+{\hat{I}}_{{\rm{\min }}})$ is given by

5 $$\frac{{V}_{{\rm{red}}}}{{V}_{0}}=|{J}_{0}(2\pi \frac{{A}_{{\rm{eff}}}}{{p}_{{\rm{G}}2}})|\mathrm{.}$$

Thereby, it was taken into account that the mean intensity at the former phase-step position of the maximum may become the minimum and vice versa by taking the absolute value. The effective amplitude *A*_eff_ depends on the phase *ϕ*_osc_ between the oscillations of *G*0 and *G*1. The worst case scenario is given for *ϕ*_osc_ = *π* meaning both oscillations occur in opposing directions increasing the vibration amplitude. Assuming oscillation amplitudes of *A*_*G*0_ = *A*_*G*1_ = 0.2 μm for each grating and considering the magnification, the effective amplitude is calculated by

6 $${A}_{{\rm{eff}}}=\frac{{d}_{{\rm{T}}}}{l}({A}_{G0}+{A}_{G1})+{A}_{G1},$$

resulting in *A*_eff_/*p*_2_ = 0.077 for the prototype.

### Data availability

The datasets generated during and/or analyzed during the current study are available from the corresponding author on reasonable request.
